# Walnut Shell Pretreatment in Regard to Its Combustion Properties

**DOI:** 10.3390/ma18061208

**Published:** 2025-03-08

**Authors:** Michał Nabiałczyk, Agnieszka Bala-Litwiniak, Dorota Musiał, Arkadiusz Szymanek

**Affiliations:** 1 Department of Production Management, Faculty of Production Engineering and Materials Technology, Czestochowa University of Technology, Armii Krajowej 19, 42-200 Czestochowa, Poland; michal.nabialczyk@pcz.pl (M.N.); a.bala-litwiniak@pcz.pl (A.B.-L.); 2 Department of Thermal Machinery, Faculty of Mechanical Engineering, Czestochowa University of Technology, Dabrowskiego 69, 42-200 Czestochowa, Poland; arkadiusz.szymanek@pcz.pl

**Keywords:** biomass boiler, calorific energy, chemical analysis, combustion, drying process, flue gas, walnut shell, waste biomass

## Abstract

Shortages in the energy market for traditional fuels, rising prices, and the requirements placed on member states by the European Union to reduce greenhouse gas (GHG) emissions are resulting in an increased interest in alternative energy sources. One such source is waste biomass. This biomass is not only ecological and publicly available, but, unlike other sources of renewable energy, it is independent of weather conditions or terrain. Unfortunately, despite the enormous potential, only a few types of biomass are currently used in the energy and heating industries. To change this, a material in the form of a walnut shell that has not been used in this field before is examined. In this work, pellets made from walnut shells were analyzed for combustion in heating boilers intended for this kind of fuel, commonly used in many households. The produced pellet was subjected to a combustion process, and the emitted flue gases were analyzed to check the suitability of the fuel for the heat-generating purpose. The exhaust gas was analyzed for the presence of compounds such as CO, NO_x_, CH_4_, and H_2_S. In addition, a series of tests were conducted to determine how the drying process time and temperature of the biomass affect its subsequent heating value. As a result of this research, it was proven that the walnut shell is suitable for the production of pellets, thus obtaining high results for a calorific value of 16.90 MJ/kg, an ash content of 1.31%, and a moisture content of 8.25%. Thanks to the obtained results, it was concluded that the produced fuel can be compared with commercial pellets, as it does not differ from and even exceeds some of the values of fuels currently available on the market. The temperature and time during the drying of the biomass also showed correlations with the subsequent calorific value of the material, with a temperature of 110 °C and a time period of 90 min providing the best results.

## 1. Introduction

Traditional fuels are still used significantly around the world. Of these, the most important are fossil fuels led by hard coal, which supplies about 40–50% of the electricity and heat to various countries around the world [1,2]. Hard coal continues to be of great importance for the European Union, where its consumption in recent years has amounted to 77% compared to other primary energy carriers [3]. To put it in perspective, in 2018, the total electricity generated around the world was estimated at 26,614.8 TWh, of which hard coal alone produced almost 40% (10.101 TWh) [4]. Coal’s annual global consumption has been estimated at around 2912.7 billion tons, with its reserves standing at 891 billion tons [5]. However, the usage of the material itself is not limited to heating and power plants; instead, traditional fuels are also used by small enterprises and households [6]. For Polish households, solid fossil fuels are still dominant energy sources and amount to 92% of the usage in the energy mix [7]. This means that only 0.36 EJ out of 4.44 EJ of consumed energy in Poland in one year came from sources other than fossil fuels.

Coal supply shortages have contributed to a serious energy crisis and an increase in the price of the raw material [8]. This makes it all the more important to ensure availability and appropriate prices on the energy fuel market. This task is made more difficult due to the Russian–Ukrainian conflict and the embargo imposed by EU countries on one of the parties to the conflict, which is an important exporter of hard coal [9,10,11,12]. The effects of the outbreak of the COVID-19 pandemic also significantly contributed to the increase in energy poverty in Poland and in the European Union [13].

These economic reasons are the basis for beginning the search for alternative energy sources that could be used for heating households and small enterprises. In addition, such a search is supported by the overall energy strategy set by the European Union Commission, which calls for a 55% reduction in greenhouse gas emissions by 2030 compared to the 1990 levels and an increase in the use of renewable sources for energy production by at least 27% [14,15,16,17]. This is of great importance since the combustion process of fossil fuels for energy purposes accounts for most of the global air pollution, generating 85% of airborne respirable particulate pollution and nearly all of the sulfur dioxide and nitrogen oxide emissions to the atmosphere [18]. The long-term exposure to those gases can lead to an increase in fatal lung and cardiovascular diseases in humans and other living organisms [19].

Taking this into account, there are many examples of alternative energy sources currently used in the energy industry in Poland, such as wind, water, or solar energy [20]. However, these sources depend on unpredictable weather conditions and favorable terrain [21,22]. Therefore, we should turn to energy sources that are more independent of environmental conditions. Biomass is one such example. In Poland, it is not only an ecologically but also an economically more sound alternative due to lower VAT rates imposed on fuel made from waste biomass [23,24]. Biomass is currently used for energy purposes only to a small extent; its share compared to other renewable energy sources was only 3% in 2021 [25]. To change this state of affairs, appropriate research should be carried out on the processing and combustion of biomass, which will help determine its effectiveness as a fuel. However, before proper research can be carried out, the appropriate type of biomass must be found and collected, as the different types of biomass vary in their properties [26].

A biomass type that may be potentially useful for the energy industry is walnut. World walnut production is estimated at nearly 1.2 million tons per year, of which Turkey is the largest producer not only in Europe but also in the world, supplying about 740,000 tons of walnut annually. Italy, which is a member state of the European Union, also contributes significantly to walnut production, supplying nearly 140,000 tons per year [27,28]. Other than that, fields that cultivate walnuts for the global market are also prolific in the west part of China, Uzbekistan, South Kirgizstan, Kazakhstan, North India, Pakistan, Afghanistan, Turkmenistan, Iran, Armenia, Georgia, and the Balkans. Furthermore, the Americas are also known as the continents where walnut cultivation is performed on a large scale [29,30].

Walnuts are used primarily in the food industry, where they are used as additives in the production of sweets, desserts, and nut butters [31]. They are also processed into oil, which, in addition to the food industry, may find its uses in the production of paints and varnishes, as well as in the cosmetic and pharmaceutical industries [32,33,34]. In most cases, dried nuts are used. One of the many inconveniences of walnut production is the sheer amount of waste associated with its processing. In practice, only the seed is used and not its shell, which accounts for slightly more than half the weight of the entire not-yet-dried walnut [31,35,36,37] with moisture inside of it. Then, it is also remarkable that the moisture directly relies on the lignin content of the walnut shells, and this can impact the mechanical properties of this material [38], which is of pivotal importance to develop biomass composites with enhanced properties [39]. Consequently, the industry contributes to the production of a significant amount of waste in the form of walnut shells. They are used today in small quantities as animal bedding, an ingredient for the production of furfural in the paint industry, in the wood industry for the production of plywood, as abrasives for cleaning, and, to some extent, for the production of heat energy in domestic boilers [40,41,42,43,44]. Despite its many uses, some of the waste is arbitrarily burned in fields and orchards, wasting its energy potential [40].

The wide range of uses of the shell, including as a fuel, is not unfounded, as many authors, including Hebda et al., have stated, on the basis of their research, the possibility of effective use of the shell in the energy industry [45]. Also, Bryś et al. stated that the nutshell as a fuel can contribute to reduced environmental pollution [46]. Before burning the raw material directly, however, it must first be processed into a fuel that is not only convenient but also easy and clean to use. One affordable treatment process is palletization, which imparts certain characteristics to the selected biomass and improves its physicochemical properties [47]. Many researchers mention the favorable physicochemical properties of processed walnuts, such as their low ash value, low moisture content, and relatively high calorific value, despite being an alternative fuel [48,49]. However, as it is a fuel from waste biomass, problems related to its low bulk density are also apparent. Palletization of walnut shells can further reduce storage and transportation costs and improve combustion characteristics, resulting in cheaper, better quality, and more environmentally friendly solid fuel from waste biomass [50,51]. The advantages of pellets also include their high energy value, low ash content, combustion efficiency, and relatively high bulk density [50,52,53,54,55,56]. Before it is possible to introduce pellets to the EU or the global market, a number of studies should be conducted to verify not only the physicochemical properties of the newly developed fuel, with a calorific value at the forefront, but also the possibility of using pellets in boilers designed for this purpose.

To date, no studies have been conducted to check both the effect of drying the walnut shell on the calorific value of the material and the combustion analysis of the produced pellets. The value of such a study is further enhanced by the fact that the conventional walnut drying process is carried out at relatively low temperatures and takes a long time. The drying process can be subjected to shortening while studying its effect on the biomaterial. The study of the effect of both temperature and drying time on the heat of combustion and the calorific value of the biomaterial will be a novelty in this article. In addition, the produced pellet itself will be tested for its suitability for combustion in domestic boilers fitted for biomass fuel.

## 2. Materials and Methods

Walnut shells from the author’s personal plantations from 2022 and 2023 were used to conduct this study. The nuts were harvested by hand to avoid damaging the shells during September when most of the nuts were ripe enough to fall to the ground without human intervention. All harvested nuts were carefully cleaned of seed residue and impurities and prepared for further processing.

**Figure 1:**
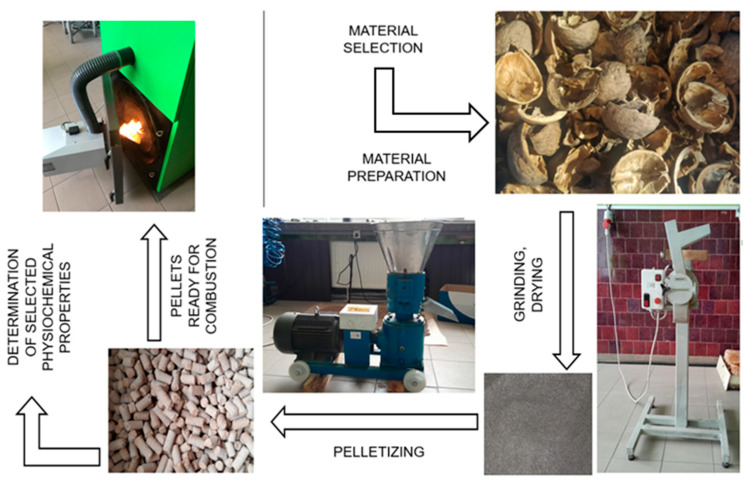
Schematic diagram of the waste biomass pelletizing process.

The walnut shells from 2022 were stored in a sheltered and heated room at a temperature not exceeding 25 °C and humidity levels between 40 and 50%, where they underwent a drying process for a period of three months. The same storage conditions were applied to the biomass from 2023. The palletization process is illustrated in Figure 1.

**Figure 2:**
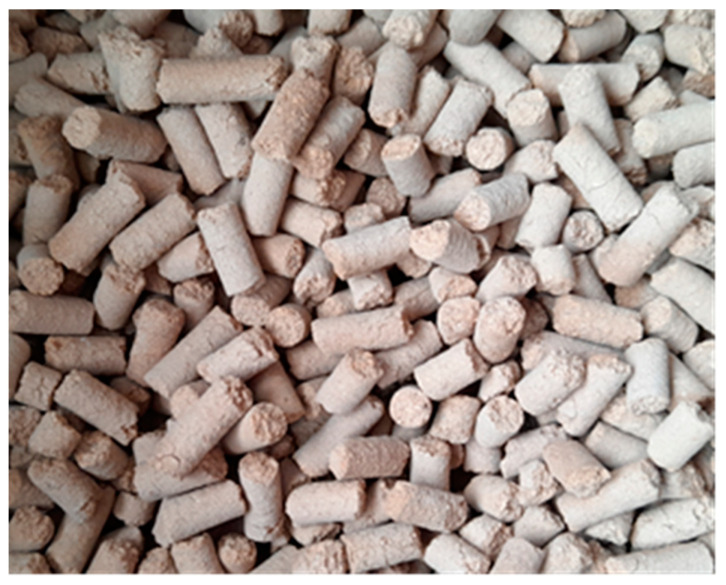
Pellets made from walnut shells.

The cleaned and dried shells were crushed using a FAEL PSM—1SRP laboratory knife mill, Testchem, Radlin, Poland and sieved through a sieve with a 1 mm hole diameter. After the biomass was crushed, the palletization process was carried out under a pressure of 1 bar using a ZLSP 150B electric pellet mill Anyang, Gemco Energy Machinery Co., Ltd., Anyang, China, with a power of 4 kW, a die with a 6 mm hole diameter, and an output of 50–100 kg/h. As a result, solid fuel was obtained in the form of pellets (Figure 2) with a length of about 3.15 to 40 mm and a diameter of 6 mm.

**Figure 3:**
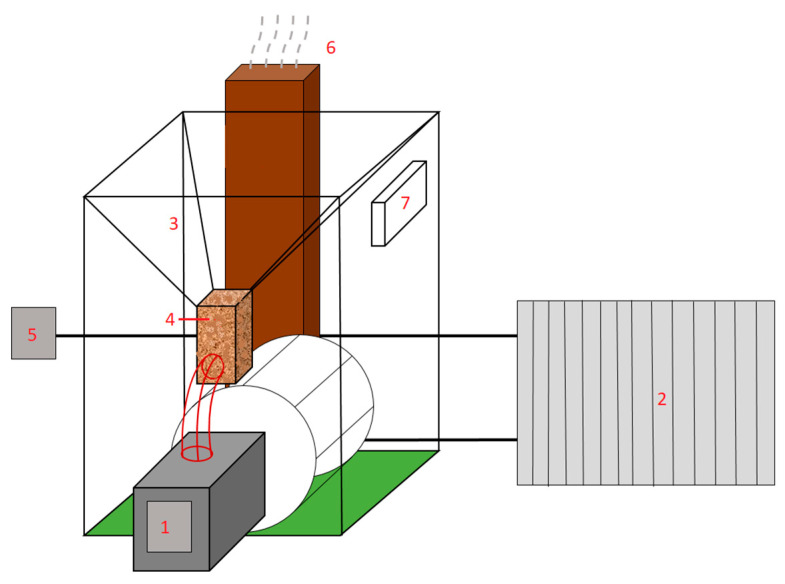
A simplified scheme of a test stand for combustion of biomass pellets. 1—burner; 2—heat exchanger; 3—fuel tank; 4—fuel feeder; 5—flue gas analyzer; 6—exhaust pipe; 7—control panel.

The palletization process used water and calcium lignosulfonate in the form of a binder in an amount not exceeding 5% by weight [57]. The pellets produced, in an amount of about 30 kg, were again dried to an air-dry state over a period of two weeks. Some of the pellets were then ground in a mortar, and a series of physicochemical property tests were conducted. The remaining pellets were used to study the combustion process in a heating boiler. A 10 kW domestic biomass boiler of the Mini Bio type (Kostrzewa, Poland), as illustrated in Figure 3, was used to burn the pellets. The boiler was equipped with a burner suitable for burning biomass in the form of pellets with a diameter of 6 to 8 mm. The inner diameter of its chamber was 0.35 m, and its total length was 0.5 m. The heat receiver is in the form of a radiator, to which water flows from a tank installed around the combustion chamber. The system was equipped with a thermostat that maintains the water within the set temperature range. The thermostat’s temperature setting during pellet combustion was set to 60 °C. In addition to changes in boiler temperature, the changes in temperature of the heated room during combustion were also measured. The combustion process included three complete cycles, where the temperature on the boiler reached the appropriate value, and the flames were extinguished. Also, the amount of fuel burned during the combustion was determined during the analysis. The combustion test was also carried out for commercial wood pellets made from coniferous tree sawdust (Pellet Karoń, DREW-PAK s.c., Koniecpol, Poland) to compare the achieved results.

During the combustion of the produced pellets, a continuous chemical analysis of the flue gases (CO and NO_x_) was carried out using a Vario-Plus analyzer (MRU, Puszczykowo, Poland), equipped with infrared sensors. The analyzer’s probe (No. 7 in Figure 3) was placed in the flue gas exhaust pipe.

The walnut shells were ground in the same way as the material intended for the pellet production. Instead of undergoing pelletization, the ground shells were separated and dried at various temperatures and time intervals in an FN 055 heating oven, ALAB, Warsaw, Poland. Temperatures of 90 °C, 110 °C, and 130 °C were used for the test, along with time intervals of 30 min, 60 min, and 90 min. The time and temperature intervals were based on the recommendations introduced from PN-EN ISO 18134-1 [58] relating to the correct determination of moisture content in solid biofuels. Determination of heat of combustion was carried out on the dried samples using a KL-12Mn2 calorimeter, PRECYZJA-BIT, Bydgoszcz, Poland in accordance with PN-EN 14918 [59].

The test was performed on the CHNS Elemental Analyzer Truspec, LECO, St. Joseph, MI, USA, which is a device for an organic elemental analysis, using the method of dynamic combustion of samples of organic and inorganic origin. The samples are burned in a reactor filled with an oxidation–reduction catalytic bed, with an electronically controlled temperature of up to 1800 °C (CHNS analyses) and in a pyrolysis reactor (O analyses). The gases during combustion are separated on a chromatographic column and then analyzed on a high-sensitivity thermal conductivity detector. The result of the analysis is the determination of the content of elements such as C, H, N, S, and O.

## 3. Results and Discussion

The outcomes of the investigation are presented in Table 1, which shows the calorific value and heat of combustion of the walnut shells with a change in drying time and drying temperature. Table 2 contains a summary of the selected physicochemical properties of these tested pellets, along with the requirements of the relevant standards.

Based on the obtained results, it can be seen that the drying process in itself significantly improves the calorific value of the potential fuel. Some significant differences in Qsa and Qia at different temperatures and time periods were observed. The highest calorific value for the walnut shells was obtained for biomass dried at 110 °C for a period of 90 min. The heat of combustion also exhibits a high value for this range, amounting to 19.96 MJ/kg. Similarly high results were obtained using a temperature of 90 °C and a time of 90 min, where Qsa and Qia reached 19.74 MJ/kg and 18.51 MJ/kg, respectively.

The lowest values were characterized by a sample that was dried for 90 min at 130 °C, reaching 18.05 MJ/kg for the calorific value and 19.28 MJ/kg for heat of combustion. The sample dried at 110 °C for one hour, which is closest to the requirements of the European standard (PN-EN ISO 18134-1), was characterized by a calorific value of 18.40 MJ/kg, thus confirming the effectiveness and validity of the temperature and time intervals commonly used. Upon analyzing the other ranges, it can be observed that a further increase in the drying time at a temperature of 130 °C results in a decrease in the obtained values of Qsa and Qia.

It can be concluded from the analyzed results that the drying process significantly affects the calorific value of biomass and, consequently, translates into the combustion process of the fuel produced from it in itself. Also, the time and temperature intervals by themselves affect the change in calorific value to some extent. As the results show, a higher temperature and a longer drying time of the material do not necessarily have a positive effect on this characteristic. A drying process that is too long or too short can lead to a deterioration of the calorific value and heat of combustion. Also, an improperly selected temperature that is too low or too high can contribute to the deterioration of potential fuel properties. Therefore, it is necessary to choose these two drying parameters so that the calorific value reaches the highest possible value. For the walnut shell tested, the optimal drying parameters are 110 °C and 90 min. In the combustion process, a commercial pellet with the average calorific value of 16.81 MJ/kg and a walnut shell pellet with a slightly higher Qia of 16.90 MJ/kg were utilized.

Table 3 presents the results from the CHNSO chemical analysis with a focus on walnut shell pellets and other typical biomass-based materials as reference substances. The analysis was conducted three times for each sample. Additionally, uncertainty with a t-Student distribution was applied for each data point. As can be concluded, walnut shells contain similar values of carbon, hydrogen, and oxygen and slightly more nitrogen than the commercial biomass taken for comparison. “Other” content was not identified in the CHNSO analysis and represents residual water and mineral content.

**Figure 4:**
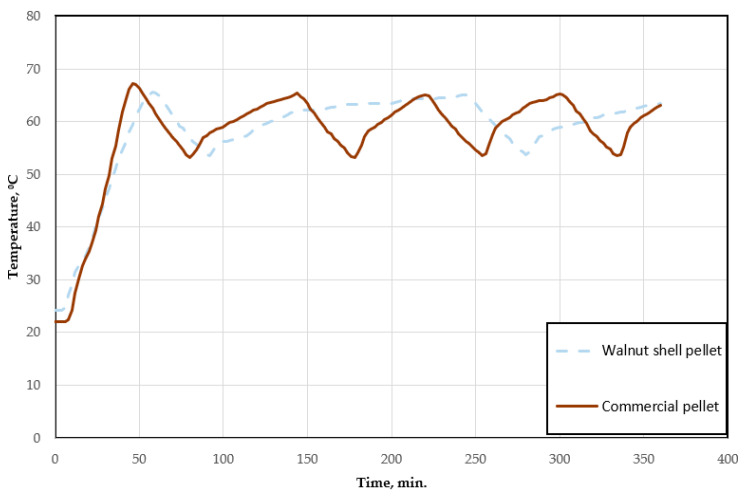
Combustion temperature history.

**Figure 5:**
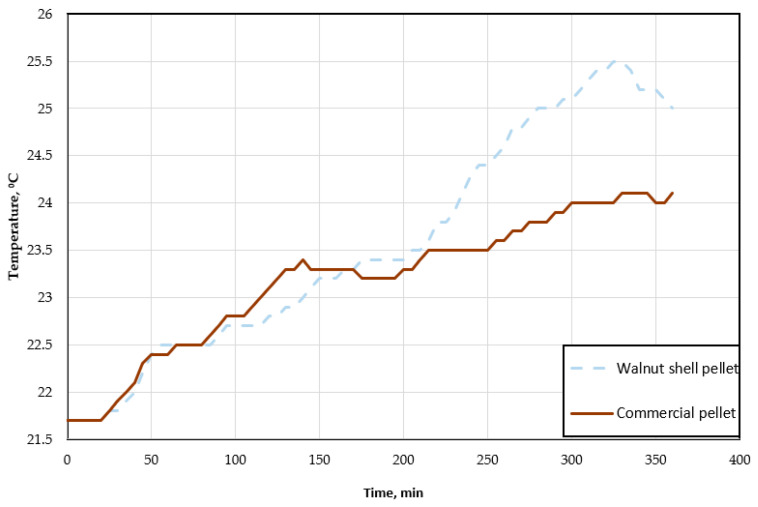
Heated room temperature history.

The combustion process of the test fuel is shown in Figure 4. In this graph, the correlation between the temperature of the boiler and the combustion time can be observed for both pellets. Figure 5 shows the relationship between the temperature of the heated room and the combustion time of the test pellet and the walnut shell pellet.

**Figure 6:**
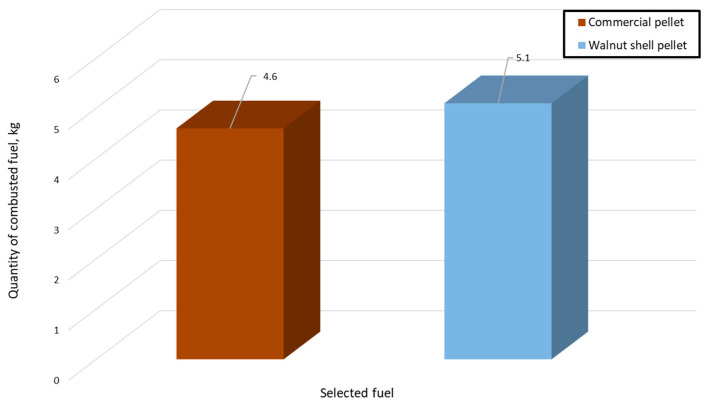
Pellet combustion process yields.

Analyzing the above graphs, the combustion process of solid fuel in the form of pellets can be observed. Both the combustion of the test pellets and walnut shell pellets proceeds similarly. Starting with an initial relatively low temperature of 23 ± 1.5 °C, the value gradually rises until it reaches approximately 65 °C. Then, the flames in the boiler are automatically extinguished, with a gradual temperature drop of nearly 12 °C. Reaching a sufficiently low value again leads to the fuel igniting and the temperature rising. This process is repeated cyclically after a certain time period. The first significant difference is immediately apparent here, which is the time it takes to reach the pre-set temperature. For wood pellets, it is a much shorter period, being less than 50 min. Walnut shell pellets do not generate a sufficiently large amount of heat, and, thus, the time for reaching the pre-set temperature is prolonged. While the initial kindling, starting from a temperature of 23 ± 1.5 °C, is the same for both pellets, subsequent igniting takes almost three times longer for pellets made from walnut shells. This is also related to the actual work of the heating boiler and the power the burner sets for itself in order to save fuel. As it reduces its power, it increases the time it will take to reach the set temperature. It takes the same amount of time to reach the second cycle for walnut shell fuel as it takes three full cycles for commercial pellets, including the beginning of the fourth cycle. The fuel consumption itself for the six-hour period is pretty close for both pellets. During this time, the combustion process uses 4.6 kg of commercial pellets. The walnut pellet required 5.1 kg over the same time period. We can, thus, observe that, despite the reduced power of the boiler, the consumption of the second fuel is higher (Figure 6).

The temperature of the heated room increased over the combustion period by 2.4 °C at a rate of 0.4 °C/h for commercial pellets and by 3.3 °C at a rate of 0.6 °C/h for walnut shell pellets. The highest temperature values reached during fuel combustion were 24.1 °C and 25 °C, respectively. These data indicate the greater amount of heat that the walnut shell pellet transfers to the heated room. This may be due to the longer cycle required to reach the set temperature. During this period, the temperature of the boiler is maintained at a higher value of about 62 °C, which makes the heat transfer process itself more intense.

**Figure 7:**
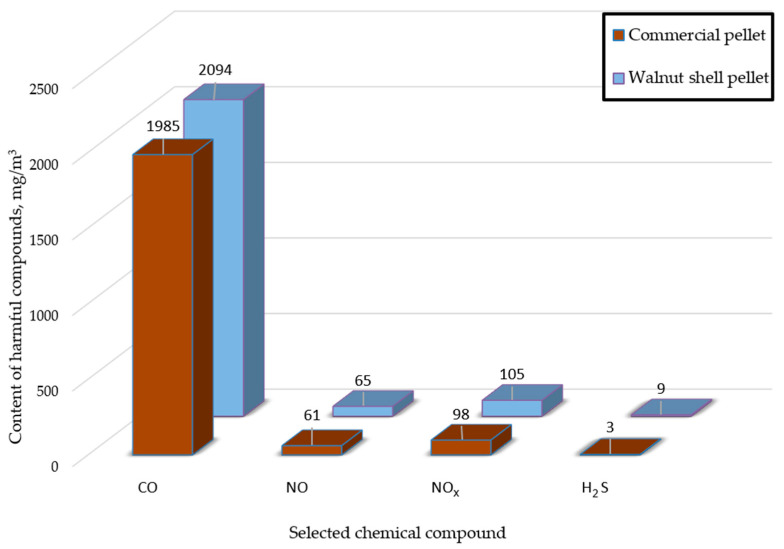
Average content of harmful substances in flue gases emitted from pellet combustion.

The results allow us to conclude that the produced fuel not only combusts in a suitable boiler but also allows heating the targeted premises in a timely manner and with modest fuel consumption. The pellet produced can, thus, compete with commercial pellets available on the fuel market today. The results of the research performed with the help of a chemical analyzer, with which the composition of the pellet’s flue gas was examined, are presented in the form of a graph in Figure 7. This graph shows the averaged content of individual harmful compounds in the flue gas from the combustion process of walnut shell pellets and sawdust commercial pellets.

From Figure 7, it can be seen that the amount of harmful compounds is relatively low in both types of fuels. The test pellet has a lower content of nitrogen oxides, carbon monoxide (II), and hydrogen sulfide than the pellet made from walnut shells. However, the difference between the fuels is slight, not exceeding 10 mg/m^3^ in the case of nitrogen oxides. The low content of nitrogen oxides is a positive occurrence, as those compounds contribute to the accumulation of greenhouse gases, acid rain, and stratospheric ozone depletion. Moreover, nitrogen oxides lead to the leaching of nitrates and, thus, deteriorate the water quality [62,63,64]. There have been a number of reports on the negative direct effects of nitrogen oxides on human health, causing cancerous changes in the human body. Moreover, the effects of these gases can cause people to experience drowsiness, depression, anxiety, suicidal thoughts, and other mental health problems [62]. Larger differences can be observed for carbon monoxide (II), but, even here, the difference in the results does not exceed 200 mg/m^3^. Carbon monoxide as a pollutant is very dangerous to human health, as it is lethal even in small concentrations and can cause varying effects from dizziness to coma [65]; therefore, a relatively low content of CO in produced fuels may improve the chances for the wider usage of fuel made from waste biomass in the heating sector. Emissions of harmful compounds from the combustion of manufactured pellets, although small, are detrimental to both human health and well-being, as well as to the state of the environment if the results are only to be compared with the other fuel contained in the work. However, the values emitted by the pellets produced are significantly lower than those resulting from the combustion of fossil fuels traditionally used in the heating industry. Fuels such as coal or lignite can emit several times higher concentrations of the aforementioned compounds, even after using appropriate equipment and processes to reduce such emissions [66,67,68]. An additional advantage that walnut shell fuel has is its low price due to the sheer nature of the material being used, that is, non-wood waste biomass. In this regard, the pellet produced is an improvement over both traditional fuels and wood biomass pellets.

## 4. Conclusions

In order to determine the suitability and combustion properties of walnut shells for energy applications, research was conducted and then thoroughly analyzed. Based on the results of the conducted investigations, the following conclusions can be formulated:

- Solid fuel made from walnut shells is suitable for combustion in domestic boilers to obtain heat.
- Pellets from waste biomass can compete with traditional pellets currently available on the energy market.
- Properly carried out, the drying process can further raise the efficiency of the fuel by increasing the calorific value of the utilized material.
- The greatest improvement to materials comes from selecting an optimal drying cycle with individually adjusted time and temperature intervals depending on the material being used. In the case of walnut shells, the most favorable condition is within a time interval of 90 min and a temperature of 110 °C.
- The elemental analysis results are comparable for both walnut shell pellets and conventional pellets, especially for CHS. Slight differences were observed for nitrogen.
- Emissions of harmful oxides and compounds during combustion of the produced fuel are comparable to that of the conventional pellets.
- The slight atmospheric pollution resulting from the use of biomass waste is outweighed by the small cost associated with the acquisition of the material and the actual process of fuel production.

To summarize, walnut shell pellets at the current stage of the research can be used as a substitute for traditional fossil fuels and wood pellets for heating households and small businesses. At the same time, a number of chromatographic studies are needed to adequately and accurately determine the potentially harmful effects of biomass in the form of walnut shells on the environment. Such tests will allow the confirmation of the presence or absence of harmful compounds, such as polycyclic aromatic hydrocarbons. The discovery of the presence of these chemicals will allow for a more in-depth assessment of the potential risks that the novelty in the form of walnut shell pellets may pose to human health and, consequently, decide the actual possibility of using such pellets as a substitute for traditional solid fuels.

In addition, micro-scale combustion calorimetry (MCC technique) and thermogravimetric analyses are being considered as additional methods for future research. Those methods could prove invaluable to assess the quality of walnut shell-based pellets more thoroughly.

Also, analyzing the effects of flue gases on the steel components of heating boilers will help to properly determine the suitability and economic value of the produced fuel. Depending on the results obtained, the fuel may not be economically viable due to the high cost of replacing or modifying heating installations.

## Figures and Tables

**Table 1:** Average calorific value and heat of combustion for dried walnut shells in relation to time and temperature.

Drying Time (min)	Drying Temperature (°C)	Analytical Calorific Value Qia (MJ/kg)	Analytical Heat of Combustion Qsa (MJ/kg)
30	90	18.28 ± 0.07	19.51 ± 0.06
	110	18.29 ± 0.13	19.52 ± 0.12
	130	18.35 ± 0.09	19.57 ± 0.10
60	90	18.33 ± 0.15	19.55 ± 0.16
	110	18.40 ± 0.02	19.62 ± 0.02
	130	18.21 ± 0.23	19.44 ± 0.22
90	90	18.51 ± 0.09	19.74 ± 0.09
	110	18.73 ± 0.12	19.96 ± 0.12
	130	18.05 ± 0.14	19.28 ± 0.14

**Table 2:** Averaged results of the selected physicochemical properties of the analyzed fuels.

Selected Physiochemical Property	Walnut Shell Pellets	Limits According to PN-EN ISO 17225-6 Standard [60]	Commercial Pellets	Limits According to PN-EN ISO 17225-2 Standard [61]
Moisture content Mad (%)	8.25	≤15	8.05	≤10
Ash content Ad (%)	1.31	≤10	1.45	≤2
Volatile content Vd (%)	77.72	-	83.51	-
Analytical heat of combustion Qsa (MJ/kg)	18.17	-	18.07	-
Analytical calorific value Qia (MJ/kg)	16.90	≥14.5	16.81	≥16.5

**Table 3:** Chemical composition (CHNSO) of selected biomass materials.

Element (%)	Walnut Shell Pellets	Commercial Pellets
C	47.80 ± 0.40	47.09 ± 0.15
H	6.20 ± 0.07	6.30 ± 0.02
N	0.92 ± 0.02	0.15 ± 0.02
S	0.1 ± 0.01	0.11 ± 0.01
O	35.41 ± 0.35	36.86 ± 0.13
Other	9.56	9.50

## Data Availability

The original contributions presented in this study are included in the article. Further inquiries can be directed to the corresponding author.
